# Cardiac Rupture and Death Following Administration of Recombinant Tissue-Type Plasminogen Activator (rt-PA)

**DOI:** 10.7759/cureus.84043

**Published:** 2025-05-13

**Authors:** Takashi Saito, Tatsuhito Ishii, Tadashi Aoki, Misato Zenko, Kiichi Yanagisawa, Yoshiro Otsuki, Ryo Sugiura, Yoshiyuki Kondo

**Affiliations:** 1 Department of Neurology, Seirei Hamamatsu General Hospital, Hamamatsu, JPN; 2 Department of Pathology, Seirei Hamamatsu General Hospital, Hamamatsu, JPN; 3 Department of Cardiovascular Medicine, Seirei Hamamatsu General Hospital, Hamamatsu, JPN

**Keywords:** autopsy, autopsy imaging, cardiac rupture, coronary artery thrombosis, pathology, recombinant tissue-type plasminogen activator, rt-pa

## Abstract

Administration of recombinant tissue-type plasminogen activator (rt-PA) in the hyperacute phase of ischemic stroke is fraught with various pitfalls. An 81-year-old man had a stroke with aphasia and right incomplete paralysis, and CT angiography showed occlusion of the left internal carotid artery (ICA) within two hours of onset. The stroke neurologist administered rt-PA and started endovascular treatment, but during the treatment, the patient went into cardiopulmonary arrest without warning and died despite resuscitation. Autopsy revealed that the cause of death was cardiac rupture due to subacute myocardial infarction in the left ventricular lateral wall. In the administration of rt-PA to patients with hyperacute cerebral infarction, special consideration should be given not only to eligibility criteria for rt-PA administration but also to screening for comorbidities.

## Introduction

The Japanese stroke guidelines allow administration of recombinant tissue-type plasminogen activator (rt-PA) to patients with hyperacute ischemic stroke with concomitant myocardial infarction if eligibility criteria are met, e.g., within four and a half hours of onset, no history of major bleeding, no hypersensitivity to therapeutic agents, no acute aortic dissection or bleeding complications, no persistent hypertension after antihypertensive therapy, no hyperglycemia or hypoglycemia, no thrombocytopenia, no abnormal coagulation capacity, and no extensive early ischemic changes [1]. However, the most serious complication reported is cardiac rupture, with a mortality rate of 64% [2]. Therefore, the use of rt-PA in patients with hyperacute ischemic stroke with concomitant myocardial infarction should be considered with caution. Here, we report a case of cardiac rupture and death after administration of rt-PA for hyperacute ischemic stroke. The cause of death was investigated in detail by autopsy.

## Case presentation

**Figure 1:**
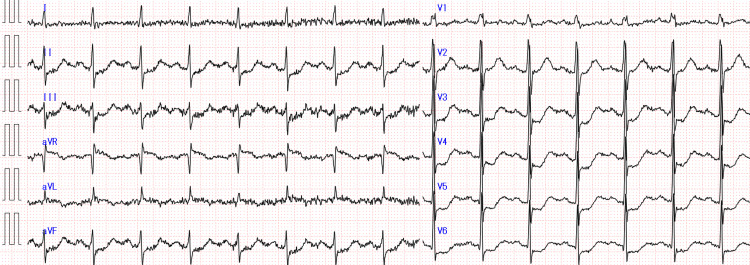
Twelve-lead electrocardiogram at the time of the visit

An 81-year-old man was brought by ambulance to our hospital two hours after his last follow-up visit with difficulty communicating and abnormal orientation of the pupil. He had hypertension in his past medical history and was admitted to another hospital two years ago with chest pain attacks, but he was discharged after refusing a coronary artery examination. Thereafter, he had no chest pain or dyspnea on exertion. His vital signs were normal. He presented with global aphasia, left-sided conjugate deviation of eyes, and right hemiparesis (a National Institutes of Health Stroke Scale score of 29). Twelve-lead electrocardiogram examination showed sinus rhythm and no atrial fibrillation (Af) but showed ST elevation in aVR and ST depression in II/III/aVF/V3-6 (Figure 1).

Laboratory findings revealed aspartate transaminase 51 international unit (IU), alanine aminotransferase 24 IU, lactate dehydrogenase 724 Unit/L, creatine kinase 356 IU/mL, creatine 1.22 mg/dL, LDL-cholesterol 143 mg/dL, hemoglobin A1c 5.7 %, D-dimer 1.1 μg/mL, and troponin I was 18,494 pg/mL when retrospectively submitted (Table 1).

**Table 1:** Laboratory findings This is the blood test result at the time of his visit to our hospital. Troponin I blood test is not a routinely performed test, but it was measured on the remaining blood sample after the event. WBC, white blood cell; RBC, red blood cell; Hb, hemoglobin; Ht, hematocrit; Plt, platelet; TP, total protein; Alb, albumin; T.Bil, total bilirubin; AST, aspartate aminotransferase; ALT, alanine aminotransferase; LDH, lactate dehydrogenase; ALP, alkaline phosphatase; γ-GT, gamma-glutamyltransferase; CK, creatine kinase; UA, uric acid; BUN, blood urea nitrogen; Cre, creatinine; FBS, fasting blood glucose; HbA1c, hemoglobin A1c; CRP, c-reactive protein

	Reference range	Value
Complete blood count		
WBC	3.3-8.6 × 10^3/μL	5.6 × 10^3/μL
Neutrocyte	41.0-79.0%	56.70%
Eosinocyte	0.3-6.0%	9.10%
Basocyte	0.3-1.4%	0.50%
Lymphocyte	21.0-51.0%	25.80%
Monocyte	3.5-8.5%	7.90%
RBC	4.35-5.55 × 10^6/μL	4.39 × 10^6/μL
Hb	13.7-16.8 g/dL	13.9 g/dL
Ht	40.7-50.1%	40.60%
Plt	15.8-34.8 × 10^4/μL	30.3 × 10^4/μL
Biochemistry		
TP	6.6-8.1 g/dL	7.7 g/dL
Alb	4.1-5.1 g/dL	3.7 g/dL
T.Bil	0.4-1.5 mg/dL	0.1 mg/dL
AST	13-30 IU/L	51 IU/L
ALT	10-42 IU/L	24 IU/L
LDH	124-222 IU/L	724 IU/L
ALP	106-322 U/L	236 U/L
γ-GT	13-64 U/L	20 U/L
CK	59-248 U/L	356 U/L
UA	3.7-7.8 mg/dL	9.9 mg/dL
BUN	8-20 mg/dL	21 mg/dL
Cre	0.65-1.07 mg/dL	1.22 mg/dL
Na	138-145 mEq/L	139 mEq/L
K	3.6-4.8 mEq/L	5.1 mEq/L
Cl	101-108 mEq/L	103 mEq/L
Triglycerides	40-234 mg/dL	163 mg/dL
HDL-cholesterol	38-90 mg/dL	47 mg/dL
LDL-cholesterol	90-140 mg/dL	143 mg/dL
FBS	73-109 mg/dL	126 mg/dL
HbA1c	4.9-6.0%	5.70%
Serology		
CRP	0.00-0.14 mg/dL	6.28 mg/dL
Troponin I	<26.2 pg/mL	18,494 pg/mL
Coagulation and fibrinolysis		
PT-INR	0.85-1.10	0.96
APTT	-	27.8 sec
D-dimer	<1.0 μg/mL	1.1 μg/mL
fibrinogen	200-400 mg/dL	591 mg/dL

**Figure 2:**
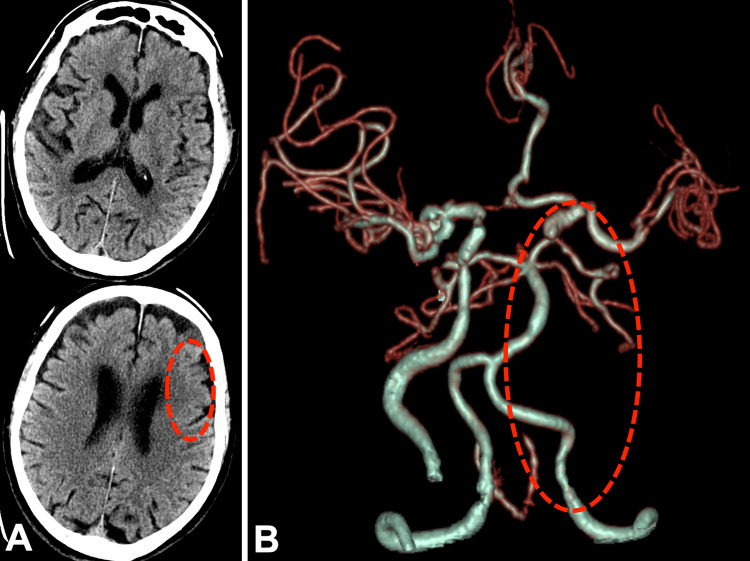
CT examination when he came to our hospital (A) CT showed no cerebral hemorrhage, with hypoabsorption in the cortical area of the left frontal lobe (inside the red dotted circle), ASPECTS of 9. (B) CTA showed occlusion of the left ICA, in other words, almost not drawn by CTA (inside the red dotted circle), and although there was collateral blood flow through the anterior communicating artery, blood flow through the left middle cerebral artery was weak. Furthermore, the left ICA was drawn to the supraclinoid segment due to collateral blood flow, suggesting the distal end of the thrombus. ASPECTS, Alberta Stroke Program Early CT Score; CT, computed tomography; CTA, CT angiography; ICA, internal carotid artery

Computed tomography (CT) showed early ischemic changes in the left frontal lobe (Alberta Stroke Program Early CT Score [ASPECTS] of 9) (Figure 2A). CT angiography showed occlusion of the left internal carotid artery (ICA) (Figure 2B).

**Figure 3:**
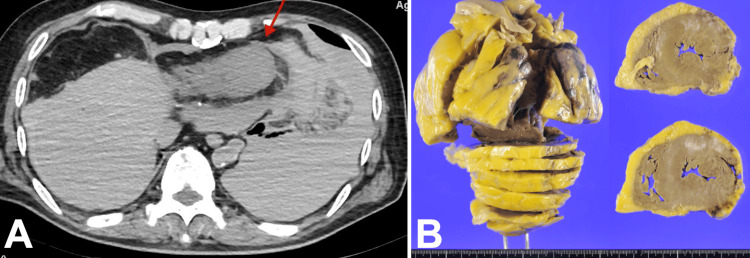
Postmortem CT imaging and gross autopsy finding (A) The apex wall was partially thinned, and there was a highly absorbing fluid accumulation in the pericardial sac to the left thoracic cavity. Hematogenous pericardial effusion and hemothorax due to cardiac rupture were suspected. (B) Gross examination of the heart revealed epicardial hemorrhage and a tear in the lateral wall of the left ventricle. Similarly, there was a slightly brownish area on the lateral wall, which was suspicious for myocardial infarction. Scattered white patches were seen on the anterior wall, reflecting areas of fibrosis and suggesting an old infarction.

Since the eligibility criteria for rt-PA administration were met, rt-PA was administered at 220 minutes after the last follow-up. Since rt-PA alone was unlikely to achieve recanalization, mechanical thrombectomy was also used to treat the large vessel occlusion. A 9Fr OPTIMO balloon-guided catheter (Tokai Medical Products, Kasugai, Japan), Sofia 7.0 aspiration catheter (Terumo Corporation, Tokyo, Japan), Marksmann microcatheter (6 × 40 mm; Medtronic, Minneapolis, MN, USA), and CHIKAI14 microwire (Asahi Intecc Co., Ltd., Seto, Japan) were used by a stroke neurologist to access the end of the left ICA occlusion. The left ICA angiography examination showed a T occlusion of the ICA top. Two approaches were performed, and partial recanalization was achieved with modified Thrombolysis in Cerebral Infarction (mTICI) classification 2a, with residual occlusion in the left middle cerebral artery. The catheter did not stray into the cardiac cavity. When the third approach was about to be performed, the patient went into cardiopulmonary arrest with a pulseless electrical activity waveform in the electrocardiogram and died despite resuscitation. Postmortem autopsy CT scan showed partial thinning of the apex wall, and a highly absorbing fluid accumulation in the pericardial sac to the left thoracic cavity, suggesting a hemopericardial effusion and hemothorax due to cardiac rupture (Figure 3A) and no apparent subarachnoid hemorrhage.

**Figure 4:**
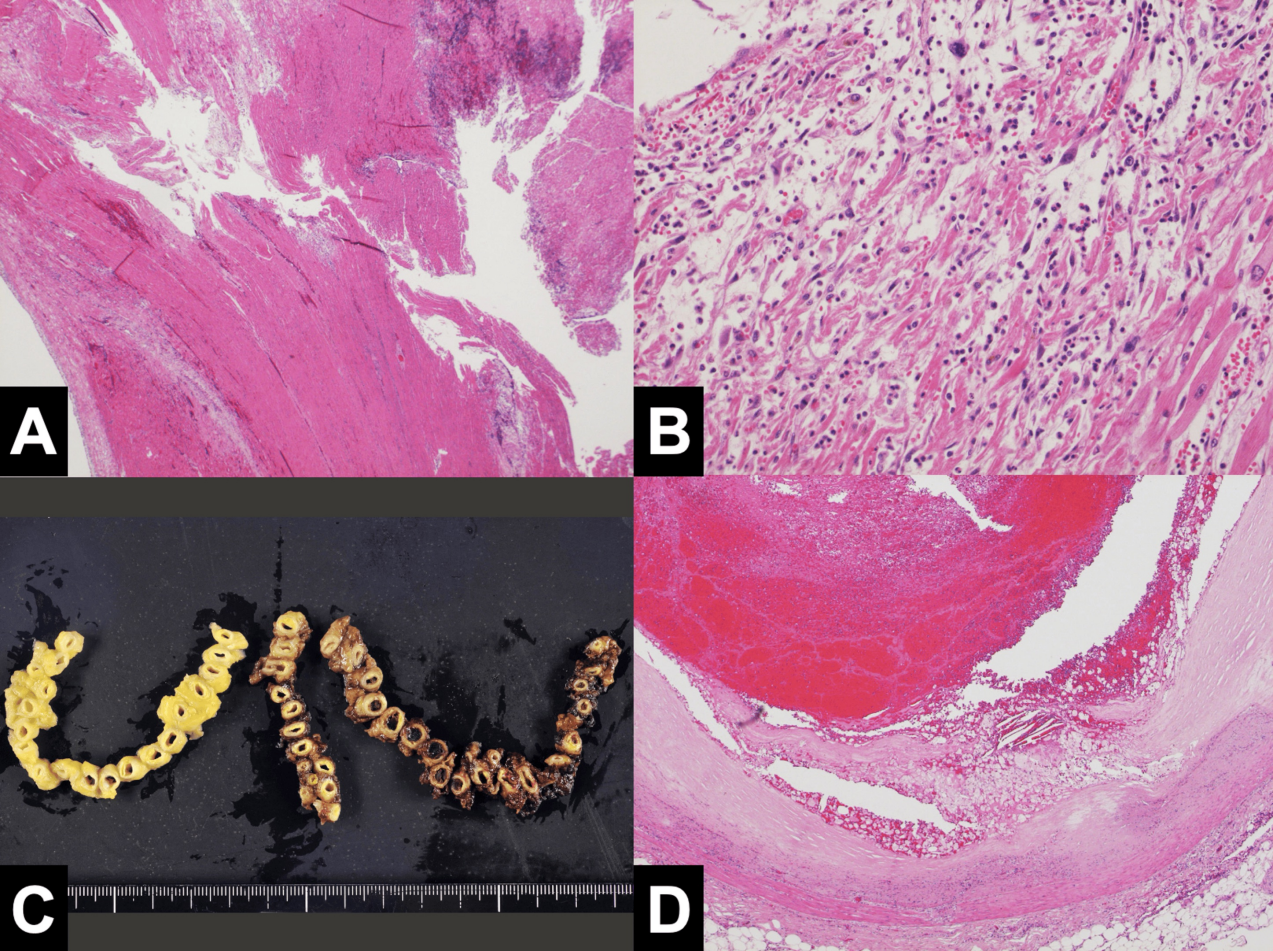
Pathological examination of myocardium and coronary artery (A) Histological examination revealed extensive coagulation necrosis around the myocardial tears and intramyocardial hemorrhage. (B) Histological examination revealed myocardial cell necrosis, histiocytic infiltration, and interstitial edema, consistent with a subacute myocardial infarction estimated to have occurred approximately one week prior to death. (C) Coronary artery cross-section showed thrombus formation in the lumen of the left circumflex artery. The images show the right coronary artery, left anterior descending artery, and left circumflex artery from left to right. (D) Histology of the left circumflex artery showed thrombus formation with rupture of an unstable plaque, consistent with a left ventricular lateral wall infarction.

Autopsy revealed myocardial infarction in the left ventricular lateral wall (Figure 3B), estimated to be around one week after onset (Figures 4A, 4B). A thrombotic embolus was found in the left circumflex artery, which was considered the responsible vessel (Figures 4C, 4D).

Cardiac rupture, mediastinal hematoma, hematogenous pericardial effusion, and hematogenous pleural effusion were observed. The patient was diagnosed with cardiac rupture and a series of changes associated with cardiopulmonary resuscitation after cardiac arrest.

## Discussion

Here, we report a case of cardiac rupture and death after rt-PA administration. The Japanese stroke guidelines allow the administration of rt-PA to patients with hyperacute ischemic stroke and concomitant myocardial infarction, if eligibility criteria are met [1]. However, one of the most serious complications of rt-PA administration is cardiac rupture, with a 64% mortality rate once it occurs [2]. Patients at high risk of cardiac rupture have been reported to include the elderly, patients with anterior wall septal infarction, and women [3]; however, in this case, being elderly was the only relevant factor.

The patient had elevations in serum creatine kinase initially and in serum troponin on retrospective evaluation (Table 1), which, together with the electrocardiographic findings, were suggestive of acute to subacute myocardial infarction at the time of presentation to the hospital. An electrocardiogram showed ST-segment elevation at aVR and abnormal Q waves, suggesting extensive anterior wall ischemia, although the QS pattern had not yet developed (Figure 1). In fact, there were no complaints of chest symptoms in the history interviewed from the family or in the physical examination of the patient, and the electrocardiogram was somewhat difficult as a non-specialist finding to suspect ischemic cardiomyopathy. Elevated creatine kinase in blood tests is a category that can be seen in rhabdomyolysis, and troponin I was not initially submitted. Furthermore, transthoracic echocardiography was not performed. In retrospect, based on the controversial findings of the electrocardiogram, noninvasive transthoracic echocardiography should be performed as a precautionary measure, and consultation should be made with a cardiologist as well. Also, if these responses increased the likelihood of ischemic cardiomyopathy, they may have been preferred over stroke intervention in some cases.

The action of rt-PA is primarily to activate plasminogen in the fibrinolytic cascade to produce plasmin, which dissolves the thrombus. And, the mechanism of cardiac rupture was considered to be that intravenous rt-PA therapy dissolved the thrombus present in the necrotic myocardium after myocardial infarction, resulting in myocardial bleeding and myocardial rupture [4]. In addition, histopathological studies have shown that the risk of myocardial rupture is high up to seven weeks after myocardial infarction due to the fragility of the tissue, but after that time, the risk is reduced due to fibrosis of the tissue. Therefore, it has been reported that it is necessary to be cautious in the administration of rt-PA for seven weeks after myocardial infarction [5]. In this case, pathologically, coagulation necrosis was widely seen around the myocardial tears in the left ventricular lateral wall, and intramyocardial hemorrhage was also observed, suggesting that myocardial hemorrhage and cardiac rupture occurred due to dissolution of the mural thrombus (Figures 4A, 4B). In addition, histiocyte leaching and fibrosis were also observed, suggesting a subacute myocardial infarction about one week after onset, a period during which the risk of cardiac rupture was high due to the fragility of the tissue.

In our discussion of the case, we considered the possibility that abnormal left ventricular wall motion as a result of recent myocardial infarction led to intracardiac thrombus and consequent ischemic stroke or that the thrombus, which had been in the intracardiac cavity for a long time, got stuck in the left main trunk or in the brain. Because patients with concomitant cerebral infarction often present with aphasia and impaired consciousness and do not complain of chest pain or other symptoms related to myocardial infarction [6], as in this case, it is more important to thoroughly confirm and evaluate simple examinations as well as to identify eligibility criteria to the administration of rt-PA.

This case highlights the possibility of myocardial infarction in patients with ischemic stroke in the early post-ischemic period, especially in the acute and subacute phases of myocardial infarction, where myocardial fibrosis has not yet developed and the patient is vulnerable. The presence of a mural thrombus may be a concern, and rt-PA administration may cause myocardial bleeding and cardiac rupture, which could be fatal. Therefore, taking into account the risk factors of individual patients, a thorough review and assessment of not only the eligibility criteria for rt-PA administration but also the patient’s own symptoms and simple examinations, such as electrocardiogram, transthoracic echocardiography, and blood test, will provide a high level of risk estimation and management for patients with hyperacute ischemic stroke and highlight the potential impact on patient outcomes.

## Conclusions

We report a case of death during treatment of hyperacute ischemic stroke after administration of an rt-PA, followed by endovascular therapy. The patient died during treatment after endovascular treatment of an ischemic stroke, which was found retrospectively to have been caused by subacute myocardial infarction, and cardiac rupture due to rt-PA administration. This case supports the warning that rt-PA administration in patients with unrecognized subacute myocardial infarction may lead to cardiac rupture and emphasizes the importance of conducting careful screening for comorbidities, especially when rt-PA is administered.
